# EphA2 and EGFR: Friends in Life, Partners in Crime. Can EphA2 Be a Predictive Biomarker of Response to Anti-EGFR Agents?

**DOI:** 10.3390/cancers13040700

**Published:** 2021-02-09

**Authors:** Mario Cioce, Vito Michele Fazio

**Affiliations:** 1 Laboratory of Molecular Medicine and Biotechnology, Department of Medicine, University Campus Bio-Medico of Rome, 00128 Rome, Italy; 2 Laboratory of Oncology, Fondazione IRCCS Casa Sollievo della Sofferenza, 71013 San Giovanni Rotondo, Italy; 3 Institute of Translational Pharmacology, National Research Council of Italy (CNR), 00133 Rome, Italy

**Keywords:** EphA2, EGFR, TKI, ephrins, CRC, CSCs, drug resistance, cetuximab, intra-tumor heterogeneity, inter-tumor heterogeneity

## Abstract

**Simple Summary:**

The Ephrin receptors and their ligands play important roles in organ formation and tissue repair, by orchestrating complex programs of cell adhesion and repulsion, however, this same system plays a role in cancer development In fact, EphA2 levels are higher in tumors vs normal tissue and further increased upon treatment, in vivo and in vitro. Changes in the molecular status of EphA2, of its subcellular localization, the absence of ligand and signals derived from the tumor context unleash the oncogenic role of EphA2 and its broad ability to promote resistance to radiotherapy, chemotherapy and targeted agents, including inhibitors of Epidermal-Growth-Factor-Receptor (EGFR). High levels of EphA2 may reduce response to cetuximab even in RAS wt CRC patients. In this work, we aim to review the current knowledge of the EphA2 function which is crucial for achieving a more effective therapeutic management of tumors resistant to EGFR inhibitors and to many other agents.

**Abstract:**

The Eph receptors represent the largest group among Receptor Tyrosine kinase (RTK) families. The Eph/ephrin signaling axis plays center stage during development, and the deep perturbation of signaling consequent to its dysregulation in cancer reveals the multiplicity and complexity underlying its function. In the last decades, they have emerged as key players in solid tumors, including colorectal cancer (CRC); however, what causes EphA2 to switch between tumor-suppressive and tumor-promoting function is still an active theater of investigation. This review summarizes the recent advances in understanding EphA2 function in cancer, with detail on the molecular determinants of the oncogene-tumor suppressor switch function of EphA2. We describe tumor context-specific examples of EphA2 signaling and the emerging role EphA2 plays in supporting cancer—stem—cell-like populations and overcoming therapy-induced stress. In such a frame, we detail the interaction of the EphA2 and EGFR pathway in solid tumors, including colorectal cancer. We discuss the contribution of the EphA2 oncogenic signaling to the resistance to EGFR blocking agents, including cetuximab and TKIs.

## 1. Introduction

### 1.1. General Structure of Eph Receptors and Ephrin Ligands

**Figure 1:**
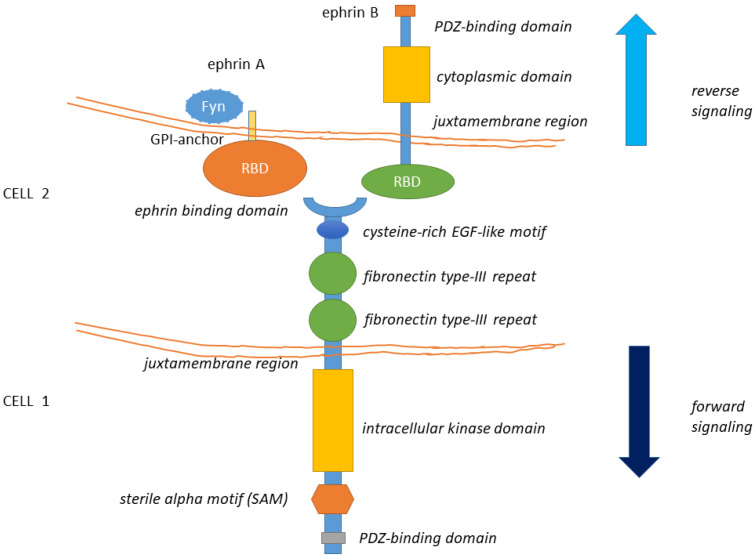
The structure of Eph receptors and their ligands is shown. Eph receptors are consisting of an extracellular structure consisting of an ephrin binding domain connected to two fibronectin type-III repeats by a cysteine-rich EGF-like motif. The juxtamembrane region connects the extracellular portion of the receptor to the intracellular kinase domain that is linked to a sterile alpha motif (SAM) domain and PDZ-binding motif. Eph ligands (ephrin-A/B) are composed of a GPI-anchored receptor binding domain in the case of the ephrin-A type and a receptor-binding domain connected by a juxtamembrane domain to a cytoplasmic domain and a PDZ interaction motif, in the case of ephrin-B. Eph-Ephrin signaling is transduced either directly (in the case of ephrin-Bs) or by interaction with Fyn (as has been observed with ephrin-As). Ligand binding likely initiates clustering, aided by receptor-receptor interactions mediated by the SAM domain and by the PDZ (Post-synaptic density protein-95, Drosophila disc large tumor suppressor (Dlg), Zona occludens-1)-domain-binding motif. The formed complexes mediate bi-directional signaling called ephrin “reverse” and Eph “forward” signaling.

The EphA2 receptor belongs to the Eph (erythropoietin-producing human hepatocellular) superfamily, the largest among tyrosine kinase receptor families [1]. Eph receptors are classified into Eph-A and Eph-B subfamilies depending on their sequence homologies and binding affinity for their cognate ephrin ligands. Although Eph receptors preferentially bind ligands of the same class, cross-binding has been described [for a review, [2,3,4] (Figure 1). All Eph receptors contain an extracellular region, with a conserved N-terminal globular ligand-binding domain (LBD), a cysteine-rich domain which comprises a Sushi and an epidermal growth factor (EGF)-like domain and two fibronectin type-III repeats (FN1 and FN2). The intracellular region contains a juxtamembrane region (JM), a tyrosine kinase domain, a sterile alpha motif (SAM) domain, and a PDZ (Post-synaptic density protein-95, Drosophila disc large tumor suppressor (Dlg), Zona occludens-1) domain-binding motif that are responsible for the interaction with effector molecules. The receptor homo- and hetero-oligomerization involves the extracellular domains (LBD and cys-rich domain) [5,6]. The SAM domain is involved in receptor-receptor interactions, possibly aiding homo- or hetero-oligomerization. The ectodomain and the intracellular domain are linked by a transmembrane helix (TM) [for reviews, [2,3] (Figure 1). Ephrins ligands are divided into ephrin-A and ephrin-B subclasses [5,7]. Ephrin-A proteins (A1–A6) are anchored to the extracellular cell membrane via a glycosyl phosphatidylinositol (GPI) linkage that could be released to activate EphA receptors at distance [8]. Ephrin-B members (B1–B3) are transmembrane proteins containing a cytoplasmic domain with several conserved tyrosine residues and a terminal PDZ-binding motif allowing the interaction with proteins involved in cytoskeleton organization and cell adhesion (Figure 1). Thus, Eph-ephrin signaling is transduced either directly (in the case of ephrin-Bs) or by interaction with intracellular proteins (like Fyn) or other transmembrane proteins (like the neurotrophin receptor p75) (as for ephrin-As [9] (Figure 1).

### 1.2. General Features of Eph-Ephrin Signaling

Since both Eph receptors and ephrins are anchored to the plasma membrane, the Eph-ephrin signaling is intrinsically bidirectional. The forward signaling is consequent to ligand binding and clustering of the receptors on the expressing cells. Trans-phosphorylation of clustered Eph receptors in the juxtamembrane domain enables efficient kinase activity [10,11]. Phosphorylation of the conserved tyrosine in the activation loop appears to be less critical for Eph receptor activation than it generally is for RTKs, mainly contributing to its maximal activity [10,12]. The reverse signaling takes place in the ligand expressing cells [13]. Both repulsive and attractive effects can be consequent to Eph-ephrin binding between cells: additionally, an initial cell-cell adhesion event mediated by the Eph-ephrin interaction may switch to a repulsive one in a time-dependent way, as the effect of cleavage of the membrane-bound ephrin or internalization of the receptor-ligand complex [14]. Ephrins can also attenuate forward signaling by Eph receptors co-expressed in the same cell [15] and also receptor delivery in extracellular vesicles to ligand expressing cancer cells has been shown to be functionally relevant, in cancer settings and in response to stress [16]. The Eph receptors also display non-catalytic functions. For instance, there are two pseudo-kinases (i.e., EphA10 and EphB6) within this large family and their function may be involved in tumorigenesis and resistance to therapy, as further discussed below for EphB6 [for a review, [17]]. Such a complexity supports a high adaptive potential, allowing for switching the Eph-ephrin signaling according to changes of both intracellular and extracellular stimuli.

## 2. EphA2 Signaling

### 2.1. EphA2 Signaling in Normal Cells

**Figure 2:**
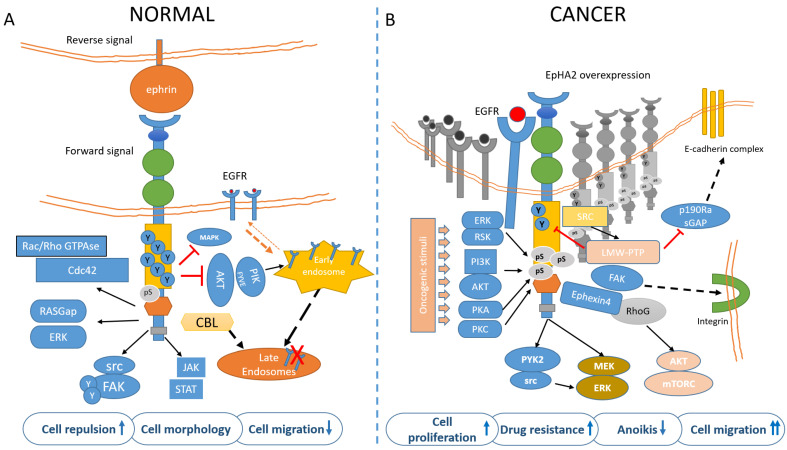
EphA2 signaling in normal (**A**) and cancer (**B**) cells. (**A**) In untransformed cells, EphA2 is engaged by its ligands, mainly EphrinA1 and highly tyrosine-phosphorylated. This mediates cell adhesion/repulsion through activation of Rac/Rho GTPAses and RASGap. Ligand binding also mediates inhibition of MAPK and AKT. Upon ligand binding, the EphA2 is targeted to endosomes in a CBL-mediated process. (**B**) In cancer cells, unliganded and overexpressed EphA2 is mainly phosphorylated in ser897 by PI3k/AKT, ERK/RSK, PKA, and PKC, in response to oncogenic stimuli The Akt-mTORC1, Raf-MEK-ERK, and Pyk2-Src-ERK signaling pathways were identified as the downstream signaling of the EphA2 non-canonical pathway. S897-phosphorylated EphA2 recruits Ephexin4 that in turn acts on RhoG to promote cell migration and anoikis resistance (this latter effect through a RhoG-AKT pathway). Further, FAK-integrin mediates cell adhesion and migration and may promote CSC features, including drug resistance (please also see Figure 3). The phospho-tyrosine content of EphA2 is also reduced by the LMW-PTPase, frequently overexpressed in cancer. The pro-tumorigenic contribution of EphA2 may thus derive from ligand independency, overexpression, reduced phospho-tyrosine content, and increased serine/threonine phosphorylation. Additionally, ligand-stimulated EphA2 negatively modulates the recycling of EGFR, by inhibiting AKT/PIKfyve, thus reducing the amount of available EGFR on the plasma membrane and migration. On the other hand, such feedback is attenuated in transformed cells, where EGFR levels in the plasma membrane are increased and this correlates with ligand independency of EphA2 and activation of motile responses to EGF.

The EphA2 receptor is a 130-kDa transmembrane glycoprotein identified in the early 90′ in Hela cells during a screen for RTKs [18]. EphA2 potentially interacts with any ephrin-A ligand, with the most frequent partner being the membrane-bound, GPI-anchored ephrin-A1, this latter discovered in 1994 [19]. After ligand binding and trans-tyrosine phosphorylation, EphA2 forms a complex with c-Cbl, to be targeted to endosomes and degraded. About 35% of the receptor is recycled back to the plasma membrane [20]. The EphA2 forward signaling is executed through ligand-instigated binding of downstream adaptors and signaling partners [21]. In fact, as for other RTKs, phosphorylation of the tyrosine residues creates docking sites for SH2/SH3 containing-proteins such as Fyn, Src, Nck, Crk, RasGAP, LMW-PTP, PI3K, and the adapter proteins Grb2, Grb10, and SLAP. EphA2 modulates cytoskeletal organization through Rho/Rac GTPases [22]. Most of these proteins affect depolymerization of the actin cytoskeleton while others modulate cell adhesion [23] with important consequences on vascular assembly, angiogenesis, and cell migration [24]. The reverse signaling elicited by EphA2 (acting as a ligand) on the ephrin expressing cell is poorly characterized and known to be mediated, for ephrinAs, by the src family kinase Fyn. Reverse signaling can mediate cell adhesion or repulsion and modulates axon guidance and synaptogenesis in the developing brain [25]. Forward signaling by EphA2 has inhibitory effects on cell proliferation, through Ras/MAPK [26]. Erk inhibition takes place through activation of GAPs and/or inhibition of GEFs [2,27]. EphA2 kinase-dependent signaling thus suppresses the AKT–mTORC1 and RAS–ERK oncogenic pathways and inhibits cell adhesion and migration [28,29] (Figure 2A). For instance, ligand-bound EphA2 attenuated Erk activation in primary keratinocytes and hepatoma cells [30]; Ephrin-A/EphA signaling suppressed Erk activation induced by IGF-1 in myoblasts, facilitating myogenic differentiation [31]. In neurons, EphA-dependent Erk inhibition suppressed the effects of the TrkB RTK on growth cone motility [32,33].

### 2.2. EphA2 in Tissue Patterning

We believe that a short journey into the role of EphA2 in tissue patterning and homeostasis may turn useful to better illustrate its involvement in tumor progression, apparently tumor-context specific but obeying signaling principles common to tissue patterning and repair. Eph receptors and ephrins have a key role in cell positioning, cell motility, cell differentiation, control of tissue morphogenesis and patterning, development of the vascular system (for a review, [2,34]). In fact, during tissue patterning, Eph receptors engagement by their ligands impedes cell mixing during tissue development and is essential to create functional topographic domains driving the formation of distinct cellular compartments [35,36,37]. For instance, forward signaling by EphA2 and its ligands aids in establishing synaptic connections in the developing nervous system by modulating growth cone guidance and axon branching [3]. Additionally, Ephrin-A2 reverse signaling inhibited the proliferation of neural progenitor cells, thus negatively modulating neurogenesis [38,39]. In developing mammary glands, EphA2 is important for promoting branching morphogenesis in vivo, as being expressed in mammary progenitor cells [40]. Ephs or ephrins may also cooperate with cell junctional modules (tight junctions and adherens junctions) to facilitate cell sorting processes and preserve the epithelial integrity and physiology in embryonal and adult tissues [41,42]. In normal colon epithelia, several studies have shown a decreasing gradient of EphB2 expression from the base to the top of the crypt, whereas EphA2 expression was observed in the differentiated compartment of the crypt apical columnar cells [43]. In fact, EphA2 is implicated in the repair of the gut epithelia [44] and of kidney epithelia during ischemia-reperfusion injury [45]. All these repair processes imply activation or reactivation of embryonal programs, like EMT or MET. Not coincidentally, as above mentioned, those programs are frequently reactivated in cancer cells [46,47].

## 3. Molecular Determinants of EphA2 Signaling in Tumors

In all the tumor settings studied, the role of EphA2, which ranges from a tumor-suppressive to a pro-tumorigenic one, depends on a number of intrinsic and extrinsic factors, some of which have been recently determined: its subcellular localization, the levels of expression, the presence of the ligand and the crosstalk with other receptors, such as the Epidermal Growth Factor Receptor (EGFR).

### 3.1. Intracellular Localization of EphA2

In non-neoplastic epithelia, EphA2 is localized to sites of cell-cell contact, in an E-cadherin-dependent way [48]. In absence of E-cadherin, associated with reduced cell-cell contacts and pro-metastatic behavior of the cancer cells, EphA2 was redistributed to membrane ruffles where it cannot engage with membrane-bound ligand ephrin-A1 on adjacent cells, thus reducing the tumor-suppressive juxtacrine signaling [41,48]. Also in cells lacking Claudin 4, another event associated with acquired pro-tumorigenic potential, EphA2 was increased and mislocalized and this correlated with increased oncogenic signaling [49].

### 3.2. Expression Levels of EphA2

EphA2 is highly expressed in many cancers with important prognostic implications. Elevated EphA2 expression positively correlated with poor prognosis, improved metastatic potential, and reduced overall survival of patients, in a tumor context-specific functioning (Table 1). Notably, the EphA2 is rarely mutated or amplified in cancer tissues [50,51]. However, expression of EphA2 may be modulated by p53, Ras and negatively modulated by estrogens [48,52,53]. In established cell line cultures, EphA2 expression was higher in cancer cells than in untransformed ones: increased staining intensity was observed, for example, in a large fraction of breast carcinoma cells (an average of 87%) when compared to benign mammary epithelial cells (an average of 3%) [54]. Related to this, overexpression of EphA2 was sufficient to transform immortalized mammary epithelial cells [55]. Additionally, EphA2 is present in GBM cells in a mainly non–tyrosine-phosphorylated state [56].

### 3.3. Ligand-Dependent EphA2 Signaling

A conspicuous amount of evidence suggests that ligand-mediated activation of EphA2 has tumor-suppressive functions. For instance, inverse expression of ephrin-A1 and EphA2 in human breast cancer cell lines was a frequent finding [80,81]. When tumors were grown in vivo, EphA2 appeared to be poorly activated by the endogenous ephrin-A [29]. Consistent with the previous observations, regulation of EphA2 expression in GBM by Fc-ephrin-A1 stimulation resulted in the loss of self-renewal ability and decreased proliferation in vitro and in vivo [82,83]. Ephrin-A1 ligand-induced EphA2 phosphorylation induces receptor endocytosis and the CBL ubiquitin-ligase mediated proteasome degradation [20,84]. Induction of ephrins may represent per se a mechanism for silencing Eph signaling. For example, during mouse ESC differentiation, FGF4 reduces EphA2 signaling, by transcriptionally inducing its ligands. This correlated with increased tyrosine phosphorylation and reduced Ser/Thr phosphorylation of EphA2 and reduced expression of pluripotency core factors, thereby leading to ESC differentiation [83]. Differently to other RTKs, activation of Eph receptors by ephrins does not increase cell proliferation or transform murine fibroblasts: conversely, it rather inhibited the Ras/MAPK and attenuated mitogen-activated protein kinase (MAPK) activation by platelet-derived growth factor (PDGF), epidermal growth factor (EGF) and vascular endothelial growth factor (VEGF), in a range of cell lines [85]. In cancer cells, including PTEN deficient prostate cancer cells and glioma cells, ephrin-dependent EphA2 activation led to rapid dephosphorylation of Akt at T308 and S473 residues leading in some cases to mTORC1 inactivation and decreased cell growth and migration [86,87,88] (Figure 2A). However, the effect of ephrin-A1 on EphA2 expressing cells may also be cell type-specific and transformation status-dependent: for instance, ephrin-A1 treatment inhibited proliferation of prostate cancer cells but failed to do so in fibroblasts [85]. Progranulin, a recently discovered EphA2 ligand, induced transient activation of MAPK in both untransformed HUVECs and transformed prostate cancer cells, but sustained activation of AKT was observed only in the latter cancer cells [89]. Altogether, this suggests that the dichotomic view (tumor suppression vs tumor promotion based on ligand availability) is too simple. Ephrin-driven forward signaling suppressed AKT activation in an ephrin-dependent way [86,87,88] ligand-bound EphA2 suppressed the recycling of EGFR to the plasma membrane, causing EGFR accumulation at the endosomes and thereby attenuating EGFR-induced cell migration. This happened in both Mouse Embryo Fibroblasts (MEFs) and in triple-negative breast cancer cells (MDA-MB-231) and was due to reduced PIKfyve activation in early endosomes following EphA2-mediated inhibition of AKT [90,91] (Figure 2A). In keeping with a tumor-suppressive role for ligand-bound EphA2, forward signaling elicited by ephrin-A ligands from normal cells on EphA2 expressing, RasV12 positive cells caused repulsion and segregation of the transformed cells [92].

### 3.4. Ligand-Independent Activation of EphA2

Low juxtacrine signaling and/or insufficient levels of ephrinA1 on cancer cells reduce EphA2 tyrosine phosphorylation [56] and this leads to attenuated internalization and degradation of EphA2 receptor, with a relative increase of EphA2 levels. Concomitantly, when the ephrinA1-mediated inhibition of AKT is removed, EGFR recycling to the plasma membrane is reduced and the EphA2 ligand-independent effect is switched on by phosphorylation on S897 (Figure 2B). Phosphorylation of the S897 residue (among the 25 ser/thr residues in EphA2) in the region linking the kinase domain with the SAM domain is the main target for “non-canonical” ephrin-independent and/or kinase-independent EphA2 signaling [87] and this activated multiple mechanisms, encompassing the downstream activation of Akt–mTORC1, Raf–MEK–ERK, and Pyk2–Src–ERK [93] (Figure 2B). Additionally, the association between EphA2 and FAK resulted in integrin-mediated adhesion, cell spreading, and migration [94] (Figure 2B). Further, unliganded, EphA2 destabilized adherent junctions via Rho-GTP activation, by inhibiting p190 RhoGAP (a Rho-GTP inhibitor) through activating the low molecular weight phospho-tyrosine phosphatase (LMW-PTP) [95]. LMW-PTP by itself may decrease the phospho-tyrosine content of EphA2 [96], possibly when activated by stress signals [97] (Figure 2B). LMW-PTP, overexpressed in many cancers, has overlapping functions with EphA2, including cell motility and resistance to therapy [98,99]. S897-phosphorylated EphA2 recruited Ephexin4 to promote cell migration and anoikis resistance via RhoG and Rac [100]. RhoG may also activate the PI3K/Akt signaling pathway to promote cell proliferation and survival independently of the activation of Rac [101,102]. RhoG-mediated activation of PI3K and Akt also suppressed anoikis [103]. Anoikis is an apoptotic modality induced by the detachment of adherent cells from the extracellular matrix and its suppression is a feature of metastatic cells [104] (Figure 2). Phosphorylation of the S897 residue in the region linking the kinase domain with the SAM domain is thus the main target for the “non-canonical” ephrin-independent and/or kinase-independent EphA2 signaling [87]. AKT, RSK, PKA, and PKC phosphorylated EphA2-S897, and this increased cell migration/invasion and metastasis and promoted cancer stem cell-like features [105,106,107] (Figure 2B). Structurally, unliganded EphA2 forms predominantly dimers rather than high-order oligomeric structures [108]. There is also evidence that the unliganded EphA2 receptor JM + kinase region may interact with phosphatidylinositol phosphates (PIPs), even if the physiological relevance of this remains to be addressed [109].

### 3.5. Tumor Context Modulates EphA2 Signaling

Besides these general mechanisms, EphA2 is endowed with tumor-context specific functions, described below. In gastric cancer cell lines, ligand-independent EphA2 activation upregulated N-cadherin and Snail, and the Wnt/β-catenin targets TCF4, Cyclin-D1, and c-Myc, thereby triggering epithelial-to-mesenchymal transition (EMT) [110]. EMT is a complex process during which tumor cells progressively acquire mesenchymal features (such as resistance to stress and acquisition of migratory ability and metabolic resilience). In detail, overexpressed EphA2 was shown to bind to Wnt-1 and to promote beta-catenin nuclear accumulation. This upregulated c-MYC that, in turn, promoted further EphA2 increase in a feed-forward manner, by binding to the EphA2 promoter [111]. As mentioned before, in breast cancer cells Ephexin4, a guanine nucleotide exchange factor (GEF) for RhoG, interacted with S897-phosphorylated EphA2 and mediated ephrin-independent cell migration, invasion, and resistance to anoikis (Figure 2B). In glioblastoma (GBM), stimulation of the cells with EGF induced MEK- and RSK-dependent EphA2 S897 phosphorylation [112]. Miao and coworkers found that EphA2 S897 phosphorylation was present mainly in grade IV human glioma specimens, in regions enriched for pS473-Akt signal and invasive cells [87]. S897 phosphorylation of EphA2 has also been involved in determining the aggressiveness of thyroid cancer cells and shown to be mediated by ERK1/2 activation downstream of oncogenes like RET (RET/PTC), KRAS (G12R), or BRAF^V600E^ [113]. The same EphA2 residue is phosphorylated by ionizing radiation in a MEK/ERK/RSK-dependent manner, mediated by increased ROS, in multiple cancer cell lines [114].

Regarding the events downstream of EphA2 S897, the Akt–mammalian target of ra-pamycin complex 1(mTORC1), Raf–MEK–ERK, and Pyk2–Src–ERK pathways were shown to be downstream effectors of the S897 EphA2 pathway in cholangiocarcinoma cells [115]. In prostate cancer and GBM, EphA2 S897 expression induced amoeboid motil-ity, which correlated with the induction of stemness markers, increased clonogenic poten-tial and tumour growth [82,116,117]. The EphA2 S897 increased in glucose starvation conditions in GBM cells and this correlated with cell survival and ROS-mediated ERK-RSK activation, induced by the cystine/glutamate antiporter xCT [118]. Thus, the S897 phosphorylation of EphA2 may work as a stress rheostat, transducing adaptive responses and thereby influencing tumor progression.

Notably, the “simple” abrogation of tyrosine phosphorylation in EphA2 may repre-sent “per se” an oncogenic signal. For instance, reintroduction of pY772A EphA2 in EphA2 knock-down naso-pharyngeal-carcinoma (NPC) cells increased cell proliferation, anchorage-independent growth in vitro and tumor growth in vivo. Mechanistically, EphA2-Y772A triggered activation (rather than inhibition) of Shp2/Erk-1/2 signaling pathway in the NPC cells, the latter involving binding of GAB1 and GRB2 as well [119]. In support of this, expression of kinase-deficient variants of EphA2 in breast cancer cells led to decreased tumor volume and increased tumor cell apoptosis [120]. 

A number of EphA2 mutations interfering with ephrin binding or kinase activity in cancer tissues such as intrahepatic cholangiocarcinoma (ICC) is being growingly recog-nized [121]. For instance, an EphA2 A859D Y772 dead mutant, exhibiting lower levels of phosphorylated Y772 and suppressed degradation through CBL, was recently identified in squamous cell carcinoma (SSC) and malignant pleural mesothelioma (MPM) speci-mens [122]. Contrariwise, tyrosine kinase activity of overexpressed EphA2 was also shown as required for the S897 phosphorylation via ERK to stimulate GBM cell prolifera-tion [112], thus showing the limit of a dichotomist view of canonical vs non-canonical EphA2 signaling and suggesting a more complex scenarios where both signaling modali-ties are highly interconnected [see also [28]]. For example, in Ewing sarcoma (ES) EphA2 promoted angiogenesis via ligand- (and caveolin-1)-dependent signaling [123], while en-hancing tumorigenicity, migration and invasion in vitro and in vivo, in an S897 depend-ent manner [124].

## 4. EphA2 Promotes Resistance to Therapy

Evidence is accumulating that the presence of a functionally competent EphA2 is re-quired for resistance to therapy. For example, whole-exome sequencing (WES) studies of paired esophageal squamous cell carcinoma (ESCC) tumors before and after radiotherapy revealed that the rate of EphA2 mutations was reduced after treatment, suggesting the need for functioning EphA2 for radio-resistance [125].

**Figure 3:**
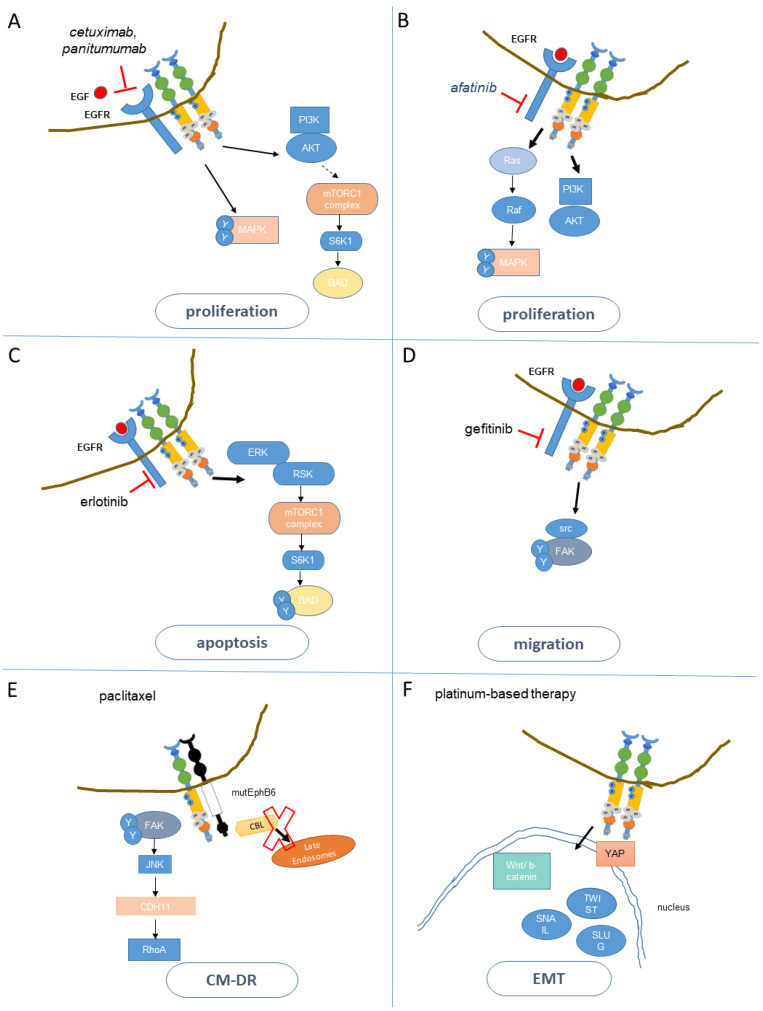
Main mechanism(s) of EphA2 mediated resistance to therapy. (**A**). EphA2-mediated activation of PI3K /AKT and MAPK mediated resistance to the EGFR blocking antibody cetuximab (and, possibly, panitumumab). (**B**–**D**) EphA2-driven activation of RSK/MTORC1/S6K1/BAD and FAK signaling mediated the resistance to anti-EGFR TKI. (**E**). Paclitaxel activated the Cell Adhesion Mediated-Drug Resistance (CM-DR) (mediated by mutEphB6-EphA2 interaction), through JNK/CDH11/RhoA/FAK signaling. (**F**)**.** Platinum-based therapies activated EphA2-mediated EMT driven by wnt-mediated activation of Snail, Slug, and Twist. Physical and functional interaction of EphA2 and YAP has been shown to mediate platinum resistance as well. Please note that while representing independent findings, it is likely that, especially for the anti-EGFR agents (**A**–**D**), the described mechanisms may coexist, given the similarity of action between these TKIs. Please also note that for the sake of clarity, in this scheme the tumor-tissue specificity has not been considered. The description of paclitaxel- and cisplatin-EphA2 driven response has been introduced here since those therapies can be used with EGFR TKI or anti-EGFR mabs in combination settings or further lines of treatment. Thin arrows indicate direct signaling, bold arrows indicate a more complex and rather indirect involvement in the indicated biological process.

The resistance to BRAF inhibitors, which is crucial for melanoma prognosis, is caus-ally associated with a mesenchymal-to-amoeboid transition (MAT) and shown as elicited by ligand-independent EphA2 activation [116,126]. In detail, the expression of active non-canonical EphA2-S897E in melanoma cells led to MAT driven by Cdc42 activation [127]. Also, ligand-independent EPHA2 signaling triggered the adoption of a therapy-induced, BRAF inhibitor resistant-metastatic melanoma phenotype through PI3K-AKT signaling [128,129,130]. In clear cell Renal Cell Carcinoma (ccRCC), Y-box binding protein 1 (YB1) mediated upregulation of EphA2 by reducing its proteasomal degradation and this strongly correlated with resistance to sunitinib and acquisition of invasive properties. Sunitinib is a broad TKI successfully used for targeting the VEGF-signaling path-way ccRCC [131]. Within this setting, EphA2 knockdown attenuated the activation of the ERK/AKT/STAT3 pathway and this reduced cell migration and metastasis in vivo, in addition to increasing sensitivity to sunitinib [132]. EphA2 overexpression was identified as required for overcoming sorafenib resistance in hepatocellular carcinoma cells: in fact, ligand mimicry in combination with sorafenib abated the resistance in vitro and in vivo by triggering EphA2 degradation [133]. Further, in gastric cancer cells resistant to oxaliplatin, EphA2 was overexpressed and this correlated with EMT features (Figure 3). EphA2 silencing restored the sensitivity to oxaliplatin by attenuating the EMT [110]. A physical and functional interaction between EphA2 and YAP was also shown to mediate resistance of gastric cancer (GC) cells to platinum in vitro and in vivo [58]. In bladder cancer cells progranulin stimulated Akt- and Erk1/2-mediated EphA2 phosphorylation at Ser897 and EphA2 depletion attenuated proliferation and cisplatin resistance [134], mimicking progranulin depletion [89].

Prenyl-binding protein phosphodiesterase-δ (PDEδ) is required for the plasma membrane association and subsequent activation of K-Ras oncogenic signaling. A kinase array in multiple K-Ras dependent cell lines showed that the resistance to PDEδ inhibitors was due to the persistent RAF/MEK/ERK signaling elicited by EphA2. In fact, concurrent blockage of EPHA2 and PDEδ inhibited the growth of the resistant cells [135]. EphA2 has also been implicated in the resistance to antineoplastic agents, such as paclitaxel, in cells bearing a mutated EphB6 pseudokinase. wtEphB6 restrained the oncogenic signaling of EphA2 and induced anoikis [136], while mutEphB6 (a missense Q926R mutation, found in lung cancers and melanomas) [137,138], promoted cell adhesion-mediated drug resistance (CM-DR) to paclitaxel. This happened by preventing CBL-mediated EphA2 degradation and by activating downstream JNK/CDH11/RhoA/FAK signaling [139] (Figure 3). In high-grade serous ovarian cancer (HGSOC) cells, a cisplatin- and carboplatin-induced ERK1/2-RSK1/2-EphA2-GPRC5A signaling mediated acquired chemoresistance in vitro and in vivo. Inhibition or knockdown of RSK1/2 strongly reduced EphA2-S897 phosphorylation in favor of the ligand-induced tumor-suppressive tyrosine phosphorylation, thus triggering downregulation of EphA2 and reverting platinum resistance of the ovarian cancer cells [140].

The effect of EphA2 on tumor resistance is also mediated by its physical and functional interaction with ErbB2/EGFR and the subsequent activation of signaling pathways that involve Ras/MAPK and RhoA [141]. In vivo, chronic trastuzumab treatment resulted in the phosphorylation of EphA2 through Src kinase, causing the activation of PI3K/Akt and MAPK pathways [142]. Last but not least, EphA2 has also been identified as a tumor intrinsic driver of immunosuppression in pancreatic adenocarcinoma, possibly through modulating the levels of the cyclooxygenase-2 COX-2 [143].

### EphA2 and CSCs

It is possible that part of the contribution of EphA2 to therapy resistance may be attributed to effects on CSCs. CSCs are cancer cell subpopulations endowed with great adaptive potential which allows them to survive therapy-induced stress and to drive tumor relapse and metastasis, as an effect of molecular adaptive processes fueling clonal rearrangements of cancer cell subpopulations. CSCs are characterized by the expression of stemness genes and detoxifying systems and by great metabolic plasticity [144,145,146,147]. Epithelial to mesenchymal (EMT) transition is believed to play a role in fueling the emergence of CSC subpopulations [148]. Recently, EphA2 has been found to play an important role in many aspects of EMT, including induction of a mesenchymal-like phenotype, inhibition of epithelial characteristics, and crosstalk with EMT-related signal transduction pathways, such as the previously described E-cadherin, RAS/MAPK, and Akt/mTOR networks [149]. Similarly, EphA2 was shown to suppress anoikis, this latter an important feature of CSCs [100]. In keeping with the potential for EphA2 to impinge on cancer cell stemness features, several observations point to a role for EphA2 in promoting survival and functions of those cell subpopulations. For instance, in human GBM, a comparison between EphA2^low^ and EphA2^high^ populations indicated that the EphA2^high^ population can maintain self-renewal property and tumorigenicity. In an orthotropic murine xenograft model, mice with tumors of high EphA2 expression exhibited shorter survival than those of low EphA2 expression [82]. EphA2 was shown also to promote growth, invasion, and survival of non-small-cell lung cancer (NSCLC) stem cell-like populations (enriched for the Aldehyde Dehydrogenase activity, ALDH) [150] by increasing the levels of p-JNK. Depletion of EphA2 in purified ALDH^positive^ cells markedly inhibited their tumor-forming ability in vivo [151].

## 5. The EGFR-EphA2 Crosstalk in Cancer: Partners in Crime

Colocalization of EGFR with EphA2 in cancer cells was shown [152] together with modulation of adhesion-induced EphA2 expression by activated EGFR [153]. In vitro experiments have suggested that EGFR is important for both EGF and ephrinA1-induced EphA2 phosphorylation and that activated EGFR can phosphorylate EphA2 in the absence of ligand. Thus, unliganded EphA2 acts as a downstream effector of EGF receptors to promote cancer cell motility and invasion [55,80,87,141,152]. In detail, EGFR, in multiple cancer settings, stimulated the phosphorylation of unliganded EphA2 via AKT and p90RSK at S897 [87,154]. These latter effects did not require tyrosine phosphorylation of EphA2 [154]. Further, as before mentioned, unliganded EphA2 can increase the EGFR plasma membrane presence by attenuating ephrin-driven AKT-mediated inhibition and in turn promoting further S897 phosphorylation by EGFR [90]. Transcriptionally, EphA2 is a target of Ras-MAPK signaling [155] and was expressed at elevated levels in epithelial cells expressing oncogenic RasV12 through MEK-ERK activation [80].

### 5.1. Resistance to Anti-EGFR Agents

The development of anti-EGFR agents has changed significantly the prognosis of patients affected by EGFR-dependent solid tumors, including NSCLC and CRC patients. These agents currently include both monoclonal antibodies which block ligand-induced acti-vation of EGFR (cetuximab, panitumumab) and EGFR tyrosine kinase inhibitors (TKI) (for a review, [156]). In detail: cetuximab and panitumumab induce EGFR internalization and degradation after binding to the external domain of EGFR [157,158]. Quickly arising re-sistance is the major challenge affecting the efficacy of these anti-EGFR mAbs [156]. Ras mutations interfering with the Ras GTPase activity, which increase activation of PI3K/AKT and MAPK downstream pathways in an EGFR-independent way, are among the main determinants of this resistance. Notably, a low proportion of cancer cells with mutated Ras is sufficient to confer a collectively resistant phenotype in tumors under ce-tuximab or panitumumab treatment [159]. It is estimated that 40% of CRC patients bear K-Ras mutations and are non-responsive to cetuximab [160,161]. Additionally, 25% more of patients respond poorly to anti-EGFR agents, partially because of additional mutations in BRAF, NRAS and PIK3CA [162]. Last but not least, about 30% of patients with no ap-parent mutations in the mentioned genes, relapse under therapy with anti-EGFR agents. There is need to identify additional determinants of cetuximab resistance.

### 5.2. EphA2 and the Resistance to Cetuximab

There is a significant amount of evidence that EphA2 plays a role in promoting or maintaining resistance to cetuximab (Figure 3). A 2013 study on 226 patients with metastatic colorectal cancer (mCRC) treated with cetuximab showed that high EphA2 mRNA receptor expression was an independent prognostic factor for poor outcome while low levels of the EphA2 mRNA were associated with objective response (OR) to cetuximab [61]. Further, treatment with recombinant ephrin-A1-Fc attenuated the unresponsiveness to cetuximab driven by MAPK and AKT activation, in N-Ras mutant and K-Ras wt metastatic CRC cells in vitro [163]. EphA2 expression was detected in >90% of NSCLC samples and positively correlated with activated EGFR but not with EGFR mutations. EphA2 expression was further increased in patients harboring K-Ras mutations and correlated with a history of smoking while predicting reduced progression-free survival (PFS) and overall survival (OS) [75]. We have studied the relationship between EGFR and EphA2 at the cell subpopulation level, by taking advantage of the murine AOM/DSS-induced adenoma to carcinoma model [43]. We found that purified EphA2^high^ cells, increased in adenocarcinoma tissue, displayed increased expression of EGFR mRNA (and lower Ephrin-A1 mRNA). Expression of the EphA2- and EGFR-related signature was prognostic in patients with stage I–III CRC. Further, in stage IV and Ras wt CRC patients, high expression of both EGFR and EphA2 was indicative of poor prognosis and poor response to cetuximab, even in Ras wt expressing patients, with only EphA2^low^ patients showing significant responses (Table 1) [62]. This is compatible with a K-Ras independent role of EphA2 in mediating the resistance to cetuximab and may be of potential therapeutic relevance for the management of K-Ras mut CRC patients.

### 5.3. Mechanisms of Resistance to EGFR TKI

The presence of EGFR activating mutations is predictive of benefit from EGFR TKIs in NSCLC, since it favors increased PFS and ORR in both first- and second-line therapy [164]. Oppositely, patients with EGFR wt tumors benefit from chemotherapy in first line settings. At least two main mechanisms have been historically shown to underlie the de-velopment of resistance to these EGFR TKI. Firstly, secondary EGFR mutations frequently arise and affect the binding of the inhibitor to the kinase domain, thus opposing the in-creased affinity for TKIs conferred by the primary mutations of EGFR [165]. For example, the T790M mutation increases affinity for ATP, resulting in loss of response to older TKIs [166,167]. Single-cell analysis showed that the simple coexistence of EGFR mut and EGFR wt cells within the tumor may attenuate the response to EGFR TKI, since only tumor cells harboring EGFR sensitizing mutations display responsiveness to TKI treatments. Further, co-occurrence of different EGFR mutations may accelerate the emergence of re-sistant clones (for a review, [168]. Secondly, EGFR-independent modulation of bypass pathways, such as PI3K and MAPK; MET amplification [169], AXL and FGFR increase [162,170,171], increased FAS/NFκB signaling [172], reduced levels of proapoptotic pro-teins, like BIM [173], all converge toward increasing resistance to anti-EGFR TKIs. Many of these mechanisms are favored by EMT, which frequently accompanies the development of EGFR-TKI resistance [174].

### 5.4. EphA2 and the Resistance to EGFR TKI

EphA2 is implicated in the resistance to EGFR TKIs, including first-, second-, and third-generation TKIs (Figure 3). EphA2 is, generally, overexpressed at the protein level, in EGFR TKI-resistant tumor cells (see below, please).

Erlotinib is a reversible, ATP-competitive inhibitor of EGFR. Loss of EphA2 reduced the proliferation of erlotinib-resistant tumor cells with the EGFR (T790M) mutation in vitro and in vivo, in TKI-resistant EGFR L858R+790M transgenic mice. The EphA2 S897expression was markedly increased in the resistant cells and also in post-relapse biopsies of four patients. Silencing of EphA2 decreased S6K1-mediated phosphorylation of the cell death agonist BAD, resulting in increased apoptosis of the cancer cells [175].

Gefitinib, a reversible small-molecule EGFR TKI, is currently used in NSCLC harboring activating EGFR mutations. Chemical proteomics studies encompassing kinase affinity purification and mass spectrometry of gefitinib resistant cell lines found EphA2 highly expressed in resistant HCC827 NSCLC cells. Notably, si-RNA-mediated knockdown of EphA2 or ephrin-A1 treatment attenuated such a resistant phenotype and reduced the levels of phosphorylated FAK, cell migration, and collagen invasion [176].

Afatinib is an irreversible pan-HER tyrosine kinase inhibitor (TKI) targeting EGFR, HER2, and HER4, currently approved for the treatment of adult patients with advanced NSCLC with activating EGFR mutation(s) or for platinum-resistant squamous NSLC. In HER2 positive GC cell lines and patient-derived-xenografts (PDXs) decreased phosphorylation of AKT, S6, ERK, MEK, JNK2, and p38-MAPK was observed after afatinib treatment. In afatinib-resistant PDXs, reactivation of the MAPK correlated with increased EphA2 mRNA and protein. The increased EphA2 was highly phosphorylated on S897. EphA2 blockade with the TKI ALW-II-41-27 reversed the acquired resistance mediated by the re-activation of MAPK thereby synergizing with afatinib [177] (Figure 3).

Altogether, EphA2 levels may prove as useful indicator in TKI-resistant tumors. A general note regarding the contribution of tyrosine kinase signaling to the EphA2-driven resistance to therapy: in some of the mentioned reports it was shown that treatment with ALW-II-41-27 could revert resistance, for instance, to erlotinib, sunitinib or cetuximab [132,175,178]. This implies the involvement of the EphA2 tyrosine kinase activity at medi-ating resistance to EGFR TKI. While the relevance of tyrosine phosphorylation for EphA2 mediated resistance cannot be excluded, we note that ALW-II-41-27 is a multi-kinase in-hibitor that inhibits EphB2, EphA3, Kit, FMS, VEGFR2/KDR, FLT1, FGR, Lyn, BMX, Bcr-Abl and also RET and SRC [179], in addition to EphA2. Some of these kinases are known to phosphorylate EphA2 on serine residues, in a ligand independent way and this correlates with EphA2 pro-tumorigenic properties. Thus, reversion of resistance by ALW-II-41-27 may not strictly indicate a requirement for inhibiting the EphA2 TK in such a process, which may rather be controlled by ligand-independent EphA2 phosphorylation in Ser-Thr residues.

In summary, the upregulation of EphA2 in EGFR TKI-treated cells confers survival advantage, by activating downstream-and parallel-pathways that overcome the EGFR inhibition. Such a scenario is made even more complex, by the EMT-promoting effect of EphA2 and its ability to crosstalk with i developmental pathways, such as the Hippo/YAP pathway. It is therefore likely that additional functions for EphA2 will be discovered in the near future. These may include, metabolic adaptation [118,180] and phenotypic repro-gramming. In fact, in a sizable percentage of lung cancer cases (10–14% cases), resistance to EGFR agents is linked to a lineage-switching phenomenon, which encompasses trans-formation of adenocarcinoma of the lung (LUAD) to squamous cell carcinoma (SCC) or small cell lung cancer (SCLC), correlating with additional mutations in p53 and Rb1. Such histotypes exhibit reduced levels of EGFR expression and this may favor resistance to TKI and (for a review, [181]). Although speculative, it is tempting to note that EphA2 has been recently shown to maintain pluripotency and restrains commitment of ESC [83]; that it has been linked to EMT induction [149,182]; that EphA2^high^ cells isolated from murine CRC do exhibit expression of stem cell markers [43,183]. It would be therefore interesting to verify the involvement of EphA2 even in this modality of resistance to EGFR TKI. 

## 6. Conclusions and Perspective

There is still a lot to learn regarding EphA2 function in cancer and its relationships to therapy-induced resistance, including that to EGFR-targeting agents. Some light has been shed on the molecular determinants of EphA2 switching from tumor suppressive to an oncogenic signaler and much information will be gained by interpreting EphA2 function in light of inter- and intra-tumor heterogeneity. Regarding the latter, we and others have shown that EphA2^high^ cell subpopulations are different in normal vs tumor tissue, in which EphA2 participates in signal cross-talk (for example, with EGFR) allowing escape from stress-induced therapy. Besides the expression levels, increased knowledge of the contribution of the posttranslational modifications of EphA2 (e.g., S897 phosphorylation) to cancer progression prompts consideration also of the post-translational status of EphA2 when searching for a predictive biomarker of response to anti-EGFR agents. In line with this, a future effort will be to understand how the unliganded, overexpressed and serine phosphorylated EpHA2 subverts its interaction spectra to exert tumorigenic function and also to identify the molecular determinants for its tumor context-specific signaling. As part of this effort, it will be important to consider the emerging connection between microRNA modulation and the emergence of CSC [184,185], given that microRNA modulation is an intrinsical part of any oncogenic signaling, including EphA2 [43]. Deeply studying those mechanisms may provide important insights and may aid the search for actionable downstream targets and for biomarkers useful for patient stratification, the ultimate way to make precision therapy more effective.

## Figures and Tables

**Table 1:** Examples of EphA2 overexpression in human malignancies, with its significance and the number of cases analyzed.

Cancer Type	mRNA Protein	Linked to	Cases (*n*)	Ref
Esophageal Squamous Cell Carcinoma	Protein	loco regional metastases; pathological grade; reduced OS	80	Miyazaki et al., 2002 [57]
Gastric Cancer	Protein	cancer recurrence (in association with YAP)	47	Huang et al., 2020 [58]
Prostate cancer	Protein	pathological grading	93	Zeng et al., 2003 [59]
Colorectal cancer	mRNA protein	CSC markers (CD44 and Lgr5); reduced OS	338	Dunne et al., 2016 [60]
Colorectal cancer	mRNA	poor prognosis and response to cetuximab	226	Strimpakos et al., 2013 [61]
Colorectal cancer	mRNA	tumor progression and poor OS (EphA2 with miR-423-5p, CREB1, ADAMTS14)	1663 (TGCA)	De Robertis et al., 2018 [43]
Colorectal cancer	mRNA	worse PFS despite EGFR^high^ (cetuximab-treated patients)	80 (TGCA)	De Robertis et al., 2017 [62]
Ovarian carcinoma	Protein	aggressive features and median survival	79	Thaker et al., 2004 [63]
Ovarian cancer	mRNA protein	poor survival	118	Han et al., 2005 [64]
Epithelial Ovarian Cancer		poor survival (stronger when combined with p53null status)	79	Merritt et al., 2006 [65]
Endometrial cancer	Protein	higher pathological grade and clinical stage; shorter disease-specific survival (DSS)	139	Merritt et al., 2011 [66]
Cervical carcinoma	mRNA	decreased overall survival (OS)	206	Wu et al., 2004 [67]
Head and neck squamous cell carcinoma	mRNA protein	higher clinical stage, recurrence, and lymph node metastasis; reduced disease-free survival (DFS) and OS	98	Liu et al., 2011 [68]
Glioblastoma	mRNA protein	increased pathological grade; reduced OS	21	Liu et al., 2006 [69]
Malignant glioma	protein	decreased DFS and OS (oppositely to EphrinA1)	78	Li et al., 2010 [70]
Glioblastoma multiforme	protein	Reduced OS	40	Wang et al., 2008 [71]
Renal Cell Carcinoma	protein	increased pathological grade, reduced DFS and OS	34	Herrem et al., 2005 [72]
Renal Cell Carcinoma	protein	reduced OS	62	Xu et al., 2014 [73]
Non-Small-Cell-Lung-Cancer	protein	smoking history; reduced PFS and OS	279	Brannan et al., 2009 [74]
Non-Small-Cell-Lung-Cancer	protein	reduced overall survival (Stronger when associated with PKR)	218	Guo et al., 2013 [75]
Non-Small-Cell-Lung-Cancer	protein	brain metastases; reduced OS	270	Kinch et al., 2003 [76]
Hepatocellular carcinoma	mRNA protein	higher pathological grade; and reduced OS	40	Cui et al., 2010 [77]
Hepatocellular carcinoma	protein	decreased OS	129	Yang et al., 2009 [78]
Gastric cancer	protein	higher in high-risk macroscopic grade 3 and 4 tumors	49	Nakamura et al., 2005 [79]

## Abbreviations

Abl	abelson murine leukemia viral oncogene homolog 1
AKT	protein kinase B
Ascl2	Achaete-scute Complex Homologue 2
BRAFV600E	B-Raf Proto-oncogene, Serine/Threonine Kinase
Cdc42	cell division control protein 42 homolog
c-Myc	avian myelocytomatosis virus oncogene cellular homolog.
CRC	colorectal cancer
CSC	cancer stem cells
CTCs	circulating tumor cells
Cyclin-D1	
EGF	epidermal growth factor
EGFR	epidermal growth factor receptor
EMT	epithelial to mesenchymal transition
ERK	extracellular-signal-regulated kinase
ERK1/2	extracellular signal-regulated kinases 1/2
FACS	fluorescence-activated cell sorting
FAK	focal adhesion kinase
FGF4	fibroblast growth factor4
FGFR	fibroblast growth factor receptor
FYN	oncogene related to SRC, FGR, YES
GEFs	guanidine nucleotide exchange factors
GPRC5A	G-protein coupled receptor 5A
Grb7	growth factor receptor-bound protein 7
HNSCC	squamous cell carcinoma of the head and neck
IGF-1	Insulin-like-Growth-Factor-1
JNK	c-Jun N-terminal kinase
K-Ras	related RAS viral oncogene homolog
Krt20	cytokeratin 20
Lgr5	leucine-rich repeat-containing G-protein coupled receptor 5
LMW-PTP	low molecular weight phosphotyrosine phosphatase
mAb	monoclonal antibody
MAPK	map kinase
MEK	mitogen-activated protein kinase
MET	mesenchymal to epithelial transition
mTOR	mechanistic target of rapamycin
N-Ras	related RAS Viral (R-Ras) oncogene homolog
PDGF	platelet-derived growth factor-beta
PDGFR	platelet-derived growth factor-beta receptor
PI3K	phosphoinositide 3-kinase
PIK_FYVE_	Phosphoinositide Kinase, FYVE-Type Zinc Finger Containing
PKA	protein Kinase A
PKC	protein Kinase C
PTEN	Phosphatase and Tensin Homolog
PYK	proline-rich protein tyrosine kinase 2
Rac1	ras-related C3 botulinum toxin substrate 1
Ras-GAP	Ras-GTPase-activating protein
RET (RET/PTC)	REarranged during Transfection
Rho-GEFs	Rho Guanidine nucleotide Exchange Factors
Rho-GTP	Rho family of small GTP-binding proteins
RSK	ribosomal protein S6 kinase
siRNAs	antisense oligonucleotides
Src	proto-oncogene tyrosine-protein kinase
TCF	T-cell factor
TCF4	T-cell factor 4
TKI	tyrosine kinase inhibitors
TKR	tyrosine kinase receptor
VEGF	vascular endothelial growth factor
xCT	cystine/glutamate exchange transporter
